# Accuracy between 2D Photography and Dual-Structured Light 3D Facial Scanner for Facial Anthropometry: A Clinical Study

**DOI:** 10.3390/jcm12093090

**Published:** 2023-04-24

**Authors:** Rocío Cascos, Laura Ortiz del Amo, Francisco Álvarez-Guzmán, José Luis Antonaya-Martín, Alicia Celemín-Viñuela, Diego Gómez-Costa, Mónica Zafra-Vallejo, Rubén Agustín-Panadero, Miguel Gómez-Polo

**Affiliations:** 1 Department of Conservative Dentistry and Orofacial Prosthodontics, Faculty of Dentistry, Complutense University of Madrid, 28040 Madrid, Spain; fag.023.y@gmail.com (F.Á.-G.); acelemin@ucm.es (A.C.-V.); m.zafra@ucm.es (M.Z.-V.); mgomezpo@ucm.es (M.G.-P.); 2 Department of Nursing and Estomatology, Faculty of Health Sciences, Rey Juan Carlos University, 28922 Madrid, Spain; laura.ortiz.delamo@clinica.urjc.es (L.O.d.A.); joseluis.antonaya@urjc.es (J.L.A.-M.); diego.gomez@urjc.es (D.G.-C.); 3 Department of Stomatology, Faculty of Medicine and Dentistry, University of Valencia, 46010 Valencia, Spain; ruben.agustin@uv.es

**Keywords:** facial scanner, 3D, 2D, accuracy, photogrammetry, structured light, anthropometry

## Abstract

(1) Background: Facial scanners are used in different fields of dentistry to digitalize the soft tissues of the patient’s face. The development of technology has allowed the patient to have a 3-dimensional virtual representation, facilitating facial integration in the diagnosis and treatment plan. However, the accuracy of the facial scanner and the obtaining of better results with respect to the manual or two-dimensional (2D) method are questionable. The objective of this clinical trial was to evaluate the usefulness and accuracy of the 3D method (a dual-structured light facial scanner) and compare it with the 2D method (photography) to obtain facial analysis in the maximum intercuspation position and smile position. (2) Methods: A total of 60 participants were included, and nine facial landmarks and five interlandmarks distances were determined by two independent calibrated operators for each participant. All measurements were made using three methods: the manual method (manual measurement), the 2D method (photography), and the 3D method (facial scanner). All clinical and lighting conditions, as well as the specific parameters of each method, were standardized and controlled. The facial interlandmark distances were made by using a digital caliper, a 2D software program (Adobe Photoshop, version 21.0.2), and a 3D software program (Meshlab, version 2020.12), respectively. The data were analyzed by SPSS statistical software. The Kolmogorov–Smirnov test revealed that trueness and precision values were normally distributed (*p* > 0.05), so a Student’s *t*-test was employed. (3) Results: Statistically significant differences (*p* ≤ 0.01) were observed in all interlandmark measurements in the 2D group (photography) to compare with the manual group. The 2D method obtained a mean accuracy value of 2.09 (±3.38) and 2.494 (±3.67) in maximum intercuspation and smile, respectively. On the other hand, the 3D method (facial scanner) obtained a mean accuracy value of 0.61 (±1.65) and 0.28 (±2.03) in maximum intercuspation and smile, respectively. There were no statistically significant differences with the manual method. (4) Conclusions: The employed technique demonstrated that it influences the accuracy of facial records. The 3D method reported acceptable accuracy values, while the 2D method showed discrepancies over the clinically acceptable limits.

## 1. Introduction

The analysis of facial soft tissues is critical in many fields of dentistry, especially in prosthodontics, orthodontics, and surgery. Qualitative and quantitative assessment of facial appearance and function is of great importance for a complete diagnosis and good treatment planning [1]. Due to the constant improvement of technology in daily dental clinical practice, many tools can be used to achieve this purpose, such as 2D and 3D methods.

In the past, facial measurements were taken with a caliper directly on the patient’s face. Nevertheless, this method has some limitations, such as data storage, lack of practicality, difficulty in communication with the laboratory, and difficult clinical applicability [2,3]. It has meant that today, 2D and 3D methods are the most widely used alternatives for this purpose. The facial references obtained through these systems can be integrated with other digital technologies such as intraoral scanners (IOSs) and computer-aided design (CAD) software programs to enable accurate diagnosis, treatment planning, and long-term follow-up of the patient. This integration allows for multiple measurements to be taken in the absence of the patient, ensuring the reliability and consistency of the data [1,2]. Thus, digital methods are more practical, saving time for both the patient and the operator.

Two-dimensional methods, such as photography, are susceptible to errors due to the influence of perspective and the lack of three-dimensional information [2,4]. In fact, in esthetic dentistry, photography does not offer all the information necessary to analyze the smile, the relationship of the teeth with the lips, and the face [5,6]. To carry out the Digital Smile Design (DSD) protocol, it is necessary to make a video or a 3D record that captures both a static and dynamic smile since the dental parameters vary according to the dynamics of the lips [6,7]. The information obtained through 3D methods will allow facial integration in rehabilitation planning, improving communication with the patient and the laboratory technician, as well as increasing the predictability of the treatments [6,7].

Facial scanners allow facial reconstructions in a single procedure with high-resolution color and without radiation [8,9,10,11,12]. However, they also have limitations, as they can sometimes present distortions of the recorded volume [8,13]. The distortions generated and the lack of accuracy may vary depending on the different technologies used by the facial scanner to generate 3D reconstructions, including lasers, photogrammetry, stereophotogrammetry, and structured light [8,12,13].

According to International Organization for Standardization (ISO) 5725-1, the term “accuracy” is defined as a combination of precision and trueness. Trueness is the closeness of the measurement results to the true value, while precision is the repeatability or reproducibility of the measurement obtained under the same conditions [14]. The accuracy and preference between the integration of 2D and 3D facial references to simulate treatment planning are unclear. This study aimed to evaluate the usefulness and accuracy of a facial scanner (using a 3D method) and compare it with a 2D method (using photography) to measure the distances between different facial anthropometric points in both the maximum intercuspation position (MIP) and smile position (SP). The null hypothesis is that there are no statistically significant differences in accuracy between the different methods studied, and there are no significant differences in the distances between MIP and SP measured by the different methods. 

## 2. Materials and Methods

### 2.1. Participant Selection

Sixty participants were recruited for this clinical trial. All the participants were engaged in the university environment (undergraduate and postgraduate students, teaching staff, and auxiliary personnel). The protocol was approved by an ethical committee and this study was performed following the principles of the Declaration of Helsinki. 

The inclusion criteria were patients over 18 years with no relevant craniofacial syndromes or deformities and no antecedents of facial or maxillofacial trauma or muscular disorders. Participants with bushy beards or physical or cognitive disabilities were excluded. In addition, it was verified that the patients did not wear elements that could interfere with the records, such as glasses or piercings, and that the forehead was clear (hair up). 

### 2.2. Soft Tissue Landmarks and Linear Measurements

**Figure 1:**
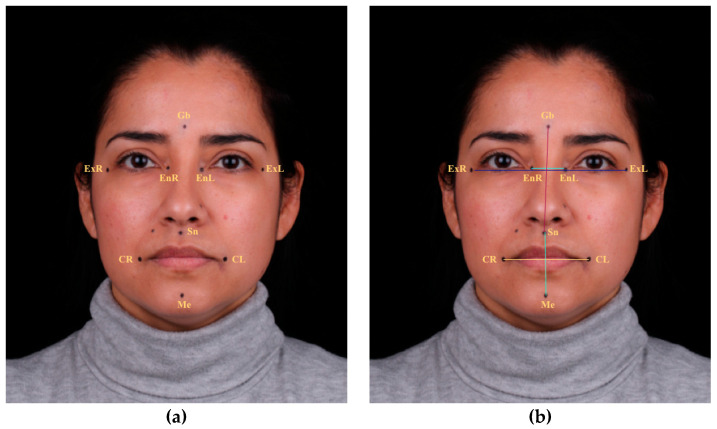
Representative frontal photographs with marked reference points. (**a**) Anthropometric landmarks: glabella (Gb), internal endocanthion of the right eye (EnR), internal endocanthion of the left eye (EnL), external exocanthion of the right eye (ExR), and external exocanthion of the left eye (ExL), subnasal (Sn), right chelion (CR), left chelion (CL), and menton (Me). (**b**) Interlandmaks distances: subnasal–glabella (Sn–Gb, red line), subnasal–menton (Sn–Me, green line); between inner edges of the eyes (EnR–EnL, light blue line); between outer edges of the eyes (ExR–ExL, dark blue line); and intercommissural (CR–CL, yellow line).

Two independent operators, F.A.G. and L.O.D.A., who were previously trained in the use of the 2D and 3D systems, identified and marked nine anthropometric soft-tissue landmarks (Table 1). They used a black indelible marker (Permanent Lumocolor F, Staedtler, Mars GmbH & Co. KG, Nuernberg, Germany) and a 15 cm ruler to ensure parallelism between the marked points. The anthropometric references considered were the glabella (Gb), internal endocanthion of the right eye (EnR), internal endocanthion of the left eye (EnL), external exocanthion of the right eye (ExR), external exocanthion of the left eye (ExL), subnasal (Sn), right chelion (CR), left chelion (CL), and menton (Me), maintaining the same points during all the data collection (Figure 1). 

The independent operators (F.A.G. and L.O.D.A.) were instructed, calibrated, and supervised by another operator (R.C.), who made sure that all the measurements were taken following the same methodology.

The linear distances registered were: subnasal–glabella (Sn–Gb), subnasal–menton (Sn–Me); between inner edges of the eyes (EnR–EnL); between outer edges of the eyes (ExR–ExL); and intercommissural distance (CR–CL) (Table 1 and Figure 1). The data were collected by two independent operators (F.A.G. and L.O.D.A.) using three different measurement methods: clinically on the patient’s face with a digital caliper (the manual group, considered the control group), digitally on 2D photographs (2D group), and digitally on a 3D facial scan (3D-AFT group). A single measurement for each linear distance was performed for each position (maximum intercuspation position (MIP) and smile position (SP). The direct method or manual group was considered the reference or “gold standard” as in previous studies [1,3,8,9].

### 2.3. Measurements Conditions

The ambient lighting conditions were standardized during the data collection procedure. The methodology was performed in a clinical room with no windows and fluorescent artificial ceiling lights. A controlled illuminance of 1000 lux (LX1330B Light Meter; Dr. Meter Digital Illuminance, Shenzhen, China) was established following the recommendations for medical or examination rooms (European Lighting Standard, UNE-EN 12464.1) [15]. The participants were seated on a dental rotatable chair with their feet on the floor and their backs straight. All individuals were instructed to maintain the same posture during the whole process: natural head position, keeping the eyes open and looking at the horizon, and maximum intercuspation position with lips sealed, unforced, and smiling (natural, unforced smile) position. The posture of each participant was strictly controlled by the operators, and corrections were made when inadequate modifications were observed. 

### 2.4. Manual Method

**Figure 2:**
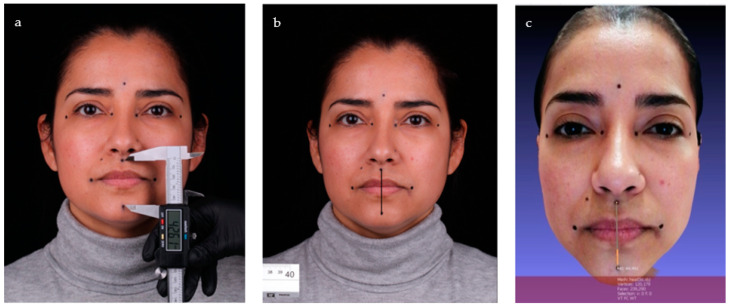
Measurement methods: (**a**) manual group using a digital caliper; (**b**) two-dimensional group with a photography program; (**c**) three-dimensional group with mesh-processing software.

For the manual group, anthropometric soft-tissue distances were measured for each participant directly with a digital caliper (IP54-Black, Qfun, China) from the center of the marked points (Figure 2a). An accuracy of 0.01 mm was reported by the manufacturer of the digital caliper.

### 2.5. Two-Dimensional Method 

For the 2D group, a digital camera (EOS80D, Canon Inc., Tokyo, Japan), equipped with a lens (EF-S 60 mm f/2.8 Macro USM, Canon Inc., Tokyo, Japan), and an external light source (36 W, 5500 K, and 55 W power, Neewer, Shenzhen, China) were used to capture a single photographic record of each patient’s MIP and SP. The same parameters were always used on the camera: shutter speed 1/125, ISO sensitivity 200, aperture f/5.6, and manual focus. All photographs taken were exported in JPG and RAW formats.

The participants were seated on a dental adjustable rotatable chair, 1.5 meters from the camera. The camera was placed in a vertical position on a tripod. Marks were made on the floor to place the chair and the tripod in the same position. 

Once all the records were obtained, they were processed on the computer and imported into a photography program (Photoshop 2020; Adobe Systems) (Figure 2b). Then, a previous pixel-millimeter calibration was performed using as a reference a ruler located at the patient’s side, included in each photograph. This procedure was repeated for all the 2D group samples. Subsequently, the linear measurements between the points determined for both positions were carried out. These distances were measured from the center of the marked points. 

### 2.6. Thee-Dimensional Method

For the 3D group, the facial scans (MIP and SP) of the participants were performed by a structured light facial scanner (Bellus 3D Face Camera PRO; Bellus 3D) connected to a tablet (MediaPad M5, Huawei, Shenzhen, China). 

The clinical scanning conditions were standardized by seating the participants in a dental adjustable rotatable chair between 30 and 45 cm away from the facial scanner in the ambient lighting conditions previously described.

The system was manipulated by the operators while the participants followed their instructions to obtain an acceptable facial scan. The 3D virtual models were exported in .obj file format. A software program (MeshLab) was used to determine the distances, placing the cross-section of the arrow at the center of each marked point (Figure 2c).

### 2.7. Statistical Analysis

The statistical analysis was performed by an independent operator. A statistical software (SPSS Statistics v 25.0 for Windows; IBM Corp.; IBM Armonk, NY, USA) was employed to compare the deviations of the anthropometric linear measurements between the control group and the test groups (2D and 3D). The Kolmogorov–Smirnov Test was used to check the normal distribution of the variables obtained. The Student’s *t*-test was employed to contrast the existing statistical significance between the variables of the different groups. The level of statistical significance was set at 5% (*p* < 0.05).

## 3. Results

Sixty volunteer participants (13 males and 47 females) aged from 20 to 44 years (mean 26, 4 years) were included in the present study. The interlandmark distance values for both positions, MIP and SP, are presented in Table 2 and Table 3, respectively (mean, maximum and minimum values, and standard deviation). The statistical normality was verified using Q-Q graphs and the Kolmogorov–Smirnov test (*p* > 0.05), which allowed the use of the parametric tests. 

**Figure 3:**
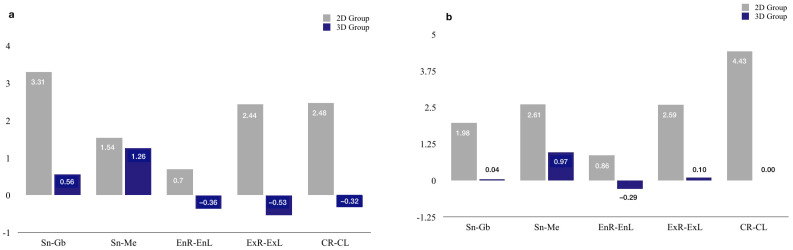
Linear deviations with the reference group for the 2D group and 3D group in maximum intercuspation position (MPI) and smile position (SP): (**a**) maximum intercuspation position; (**b**) smile.

In the 2D group, statistically significant differences (*p* ≤ 0.01) were observed in all interlandmark measurements in MIP and SP positions compared to the reference method values. In the 3D group, statistically significant differences were found in the interlandmarks Sn–Gb (*p* < 0.05), Sn–Me (*p* < 0.01), and EnR–EnL (*p* < 0.01) in MPI and Sn–Me (*p* < 0.01) and EnR-EnL (*p* < 0.01) in SP. However, no significant differences were observed in CR–CL (*p* > 0.05), ExR–ExL (*p* > 0.05) in MIP and SP, as well as Sn–Gb (*p* > 0.05) in smile (Figure 3; Table 4).

Comparing the two test groups (2D vs. 3D) by Student’s *t*-test as shown in Table 5, significant differences (*p* < 0.01) are observed in all soft-tissue interlandmarks both in MIP and SP, except for Sn-Me (*p* > 0.05) in the MIP.

## 4. Discussion

This study aimed to evaluate the usefulness and accuracy of the 3D method (a dual-structured light facial scanner) and compare it with the 2D method (photography) to obtain facial analysis in the maximum intercuspation position (MIP) and smile position (SP). Significant differences were found in the accuracy of the 2D and 3D methods as well as between the MIP and SP. Hence, both the null hypotheses were rejected. 

Literature reports a discrepancy close to 1 mm, but it is considered clinically acceptable to have deviation values up to 2 mm [10,16,17,18,19].

For the 2D group, statistically significant differences (*p* < 0.01) were observed in all facial interlandmark distances, both in MIP and SP. In MIP, the linear deviations of Sn–Me and EnR–EnL, presented values under the clinically accepted limit of 1.54 and 0.70 millimeters, respectively. In SP, it was also observed at the distances Sn–Gb (1.98) and EnR–EnL (0.86). The rest of the deviation’s magnitudes were above the clinically accepted limit. 

When evaluating the trueness of the facial scanner, the 3D group showed higher accuracy. There were no statistically significant differences (*p* ≥ 0.05) observed in the distances between the anthropometric landmarks of the ExR–ExL and CR–CL in both MIP and SP, and Sn–Gb in the SP position. However, all the linear measurements were clinically acceptable. 

When comparing the different methods, it was found that the 2D group had higher discrepancies with the reference values (3.38 ± SD mm in MIP; 3.67 ± SD mm in SP) compared to the 3D groups (2.03 ± SD mm in MIP; 1.65 ± SD mm in SP) for both MIP and SP. These values are different from other studies, where the same facial scanner showed higher accuracy (0.14 [8] and 0.32 [1]). The images captured by the 2D method reported a lower accuracy, with values of 2.09 ± SD mm and 2.49 ± SD mm in MIP and SP, respectively. On the contrary, 3D facial scans performed by the dual-structured light scanner (Bellus 3D) showed lower values in both MIP (0.61 mm) and SP (0.28 mm). In previous studies with 3D technologies, similar values were reported by Liu et al. [12], Gallardo et al. [8], and Piedra-Cascón et al. [1], who showed discrepancies of 0.61 mm and 0.91 mm, respectively (Bellus 3D). Liu et al. [12] reported values of 0.36 mm using 3D surface imaging systems (3d MD face system), which incorporate passive and active stereophotogrammetry. At last, Knoop et al. [18] reported a deviation of 0.71 and 1.33 for a white light scanner (M4D, Rodin, 4D SAS) and a structured infrared light scanner (Structure Sensor; Occipital Inc., Boulder, CO, USA), respectively. 

According to the literature, options for sample selection include using an inanimate sample, such as a mannequin, or recruiting volunteer individuals. The advantages of using an inanimate model include the simplicity of sample selection, the frequency of studies that evaluate the accuracy of scans on an inanimate model [8,12,16,20,21,22,23,24], and the avoidance of possible errors caused by involuntary facial movements and expressions of individuals, as well as the flexibility of facial soft tissue [25]. However, studies using an inanimate model may have limitations in terms of trueness and precision since individuals cannot maintain absolute inactivity like mannequins [12]. In this study, real patients were used for measurements, as one of the objectives was to evaluate how the measurements of different anthropometric points using 2D and 3D methods are affected by the contraction and relaxation of facial muscles during maximum intercuspation and smile. In the smile position, greater deviations were observed with respect to the reference group. The contraction of the muscles in this position during the acquisition of the photograph and in the digitalization of the face scan (15 s) could cause these deviations. Maintaining the same head position as well as facial expression in a smile is more uncomfortable and complex for anyone than in maximum intercuspation, which is a more relaxed and stable posture. Hence, it was crucial to ensure that the participant maintained a consistent head position and facial expression during the recording. Any changes in posture could result in significant alterations in facial morphology, leading to potential inaccuracies in both the photograph and the three-dimensional reconstruction of the face scan.

Facial measurements can be performed using anatomical structures as reference points or anthropometric facial landmarks marked with stickers or a pen [26]. Nevertheless, anatomical structures can become subjective and complex among different individuals [12,13,16,19,20,27]. Piedra-Cascón et al. [1] and Liu et al. [12] demonstrated excellent interexaminer reliability (0.99) when marking the anthropometric facial landmarks, suggesting that it is a reliable and reproducible method among different operators. The marking of the facial points to perform the measurements has been demonstrated to positively influence the accuracy [3,27] and the working time [3]. Franco et al. [3] observed a random error range of 0.65 to 2.16 mm and from 1.30 to 3.60 mm for measurements with and without the marking of the points, respectively. In the present study, an indelible marker was employed for this issue following the same methodology.

Traditionally, measurement errors in 3D face scanning systems have been evaluated with manual methods using calipers [1,2,3,10,11]. However, to avoid potential errors in human measurement, several authors [7,8,9,24,28] have utilized a software program for 3D mesh analysis that can automatically calculate discrepancies using an algorithm. 

One of the inherent disadvantages of the professional facial scanner is its cost, which promotes the discovery of a cheaper alternative. With the development of technology, the images obtained by some smartphone models can capture the face in three dimensions. The accuracy values demonstrated by several studies [24,29], in combination with their easy availability and low cost, make the use of smartphones a good alternative to facial scanners, either through applications or manual photogrammetry.

Some limitations of this study should be considered. One of them is the lack of standardized protocols for comparing the results with those of other research studies. Additionally, the reproducibility of the 2D and 3D systems and some factors, such as the analysis in different software programs and environmental conditions, were not explored. The low representativity of the male volunteer is also a limitation, as there are anatomical differences between men and women that could affect the results.

Further clinically well-designed studies are needed to increase the scientific evidence about this topic. Future studies should include the use of other facial scanner models and variations in environmental and intraoral conditions. Additionally, partially and completely edentulous subjects should be included, and the sample of participants should be more homogeneous between men and women. Moreover, the inclusion of different alignment procedures with intraoral scanning to evaluate the accuracy of the virtual 2D and 3D patient models would also be interesting. 

## 5. Conclusions

Based on the findings of the present clinical study, the following conclusions were reported:

1. The dual-structured light facial scanner (3D method) used in this study can be considered an accurate method for the reproduction of facial soft tissues, with clinically acceptable values in both relaxed and contracted facial muscle conditions.
2. The 2D method using photography was found to be inaccurate for the reproduction of facial soft tissues, as the values obtained were not clinically acceptable.

## Figures and Tables

**Table 1:** Description of anthropometric landmarks and linear distances measured.

Coding	Landmarks	Description
Sn–Gb	Subnasale–Glabella	Vertical linear measurement from glabella to subnasale
Sn–Me	Subnasale–Menton	Vertical linear measurement from subnasale to menton
EnR–EnL	Endocanthion right– Endocanthion left	Transverse linear measurement from endocanthion right and left
ExR–ExL	Exocanthion right– Exocanthion left	Transverse linear measurement from exocanthion right and left
CR–CL	Chelion right– Chelion left	Transverse linear measurement of mouth width

**Table 2:** Absolute values (mm) of the linear distances for all the groups (manual group, 2D group, and 3D group) in maximum intercuspation position (MPI).

	Manual Group			2D Group			3D Group		
Interlandmark-Measurement	Mean	Min–Max	SD	Mean	Min–Max	SD	Mean	Min–Max	SD
Sn–Gb	72.09	63.22–90.77	5.45	75.40	65.54–91.36	5.72	72.65	66.01–89.30	5.21
Sn–Me	43.04	33.59–50.71	3.54	44.57	34.84–56.11	4.13	44.29	36.94–50.70	3.15
EnR–EnL	24.53	20.40–31.42	2.28	25.22	19.87–34.61	3.19	24.17	20.70–31.32	2.28
ExR–ExL	106.30	93.22–120.23	5.66	108.74	92.17–132.01	8.54	105.77	92.47–118.95	5.52
CR–CL	60.29	45.47–71.52	4.84	62.77	49.25–78.07	6.02	59.97	49.86–71.34	4.59

SD, Standard deviation.

**Table 3:** Absolute values (mm) of the linear distances for all the groups (manual group, 2D group, and 3D group) in smile position (SP).

	Manual Group			2D Group			3D Group		
Interlandmark-Measurement	Mean	Min–Max	SD	Mean	Min–Max	SD	Mean	Min–Max	SD
Sn–Gb	68.59	61.61–83.31	4.77	70.57	61.53–86.06	5.50	68.62	61.79–83.00	4.88
Sn–Me	50.04	36.44–60.62	4.83	52.66	42.58–63.39	5.21	51.02	41.73–61.57	4.36
EnR–EnL	24.44	20.28–30.81	2.45	25.30	20.70–34.43	3.10	24.15	20.94–31.14	2.21
ExR–ExL	106.62	93.75–120.22	5.79	109.20	95.06–130.58	8.44	106.72	95.20–119.85	5.95
CR–CL	71.58	56.42–88.62	5.93	76.01	63.04–91.51	6.58	71.59	58.81–84.94	5.34

SD, Standard deviation.

**Table 4:** Linear deviations with the reference group for the 2D group and 3D group in maximum intercuspation position (MPI) and smile position (SP) (Student’s test).

	MIP						SP					
	2D Group			3D Group			2D Group			3D Group		
Interlandmark-Measurement	MSE (SD)	*t*-test	*p*	MSE (SD)	*t*-test	*p*	MSE (SD)	*t*-test	*p*	MSE (SD)	*t*-test	*p*
Sn–Gb	3.31 (3.99)	6.43 **	0.000	0.56 (1.65)	2.60 *	0.012	1.98 (3.93)	3.99 **	0.000	0.04 (1.42)	0.20 ^NS^	0.845
Sn–Me	1.54 (3.14)	3.79 **	0.000	1.26 (1.74)	5.58 **	0.000	2.61 (3.72)	5.44 **	0.000	0.97 (2.84)	2.65 *	0.010
EnR–EnL	0.70 (1.55)	3.47 **	0.001	−0.36 (0.90)	−3.07 **	0.003	0.86 (1.36)	4.91 **	0.000	−0.29 (0.99)	2.24 *	0.029
ExR–ExL	2.44 (4.74)	3.98 **	0.000	−0.53 (2.24)	−1.83 ^NS^	0.072	2.59 (4.36)	4.60 **	0.000	0.10 (2.27)	0.36 ^NS^	0.724
CR–CL	2.48 (3.49)	5.50 **	0.000	−0.32 (1.71)	−1.46 ^NS^	0.151	4.43 (4.96)	6.92 **	0.000	0.00 (2.65)	0.01 ^NS^	0.993

MSE (mean squared error); SD (standard deviation); NS, no significant; * statistically significant; ** highly significant.

**Table 5:** Comparison of interlandmark distances measured between 2D and 3D methods in maximum intercuspation position (MPI) and smile position (SP) by Student’s test.

	MIP					SP				
	MSE		*t*-Test		Effect Size R^2^	MSE		*t*-Test		Effect Size R^2^
Interlandmark-Measurement	2D-Group	3D-Group	*t*-test	*p*		2D Group	3D Group	*t*-test	*p*	
Sn–Gb	3.31	0.56	4.94 **	0.000	0.171	1.98	0.04	3.60 **	0.001	0.099
Sn–Me	1.54	1.26	0.61 ^NS^	0.544	0.003	2.61	0.97	2.71 **	0.008	0.059
EnR–EnL	0.70	−0.36	4.54 **	0.000	0.149	0.86	−0.29	5.29 **	0.000	0.192
ExR–ExL	2.44	−0.53	4.38 **	0.000	0.140	2.59	0.10	3.92 **	0.000	0.115
CR–CL	2.48	−0.32	5.58 **	0.000	0.209	4.43	0.00	6.10 **	0.000	0.240

MSE (mean squared error); NS, no significant; ** highly significant.

## Data Availability

The data presented in this study are available on request from the corresponding author.
